# Honorary Issue for Professor Anthony Fane

**DOI:** 10.3390/membranes16030077

**Published:** 2026-02-24

**Authors:** Pierre Le-Clech, Jia Wei Chew, Chuyang Tang, Anja Drews

**Affiliations:** 1 UNESCO Centre for Membrane Science and Technology, School of Chemical Engineering, University of New South Wales (UNSW), Sydney, NSW 2052, Australia; 2 School of Chemical and Biomedical Engineering, Nanyang Technological University, Singapore 637459, Singapore; jchew@ntu.edu.sg; 3 Department of Civil Engineering, The University of Hong Kong, Pokfulam, Hong Kong, China; tangc@hku.hk; 4 Department of Engineering II, School of Life Science Engineering, HTW Berlin, Wilhelminenhofstr. 75A, 12459 Berlin, Germany; anja.drews@htw-berlin.de

Professor Anthony (Tony) Fane needs no introduction within the membrane community. The large majority of the MDPI’s *Membranes* readership has already cited his work many times! For more than five decades, his pioneering scholarship, institutional leadership, and remarkable generosity of spirit have shaped modern membrane science and technology across continents. This Special Issue of *Membranes* is dedicated to celebrating his lifelong contributions as a researcher, educator, mentor, and community builder.

Tony’s scientific journey began in Coventry, United Kingdom, in 1940. After graduating from Imperial College London in 1961, he returned in 1964 to pursue his PhD in distillation, which he completed in 1968. Shortly thereafter, in 1969, he moved to Australia to join the Australian Atomic Energy Commission as a Research Scientist. His transition into academia began in 1973 when he joined the School of Chemical Engineering at the University of New South Wales (UNSW) in Sydney. Over the next decades, Tony’s intellectual curiosity and vision propelled him through the academic ranks—Senior Lecturer in 1975, Associate Professor in 1984, and full Professor in 1989, the same year he received the Whiffen Medal of the IChemE for his landmark contributions to membrane technology.

Tony’s research interests have always been broad, creative, and deeply relevant to global needs. His work spans microfiltration and ultrafiltration, membrane fouling and cleaning, reverse osmosis desalination, membrane bioreactors, novel membrane materials, hybrid systems, and the development of non-invasive monitoring and diagnostic tools. He has contributed foundational insights into membrane transport, hydrodynamics, and module design, influencing both fundamental understanding and industrial practice. To date, Tony has authored more than 500 peer-reviewed journal papers, and his work has accumulated more than 71,000 citations with an h-index of 146, achievements that underscore his extraordinary scientific impact.

But Tony’s influence extends far beyond publications. He has been instrumental in creating and nurturing two globally recognized membrane research centres. The first, the UNESCO Centre for Membrane Science and Technology (CMST) at UNSW, Sydney, Australia, was founded in 1988. From 2002 to 2006, he held the prestigious Temasek Professorship at Nanyang Technological University (NTU), before becoming the Founding Director of the Singapore Membrane Technology Centre (SMTC).

Throughout his career, Tony has also served the broader scientific community in exemplary ways. He was Editor of the *Journal of Membrane Science* from 1992 to 2005 and remains member of the Advisory Board. He has served on the boards of *Desalination* and *Reuse*, contributed to WHO’s desalination guidelines, participated in water advisory panels, and offered expertise to industry through multiple board appointments. His contributions have been recognized through numerous national and international honours, including the Esso Award of Excellence in Chemical Engineering, the Australian Centenary Medal, and honorary life membership of the European Membrane Society. He is a Fellow of ATSE, IChemE, and Engineers Australia.

Yet beyond all these achievements, what many of us cherish most about Tony is his humanity. He is unfailingly humble and unassuming, generous with his time, and committed to nurturing younger colleagues. He has a gift for listening carefully, offering thoughtful guidance, and inspiring confidence. For those fortunate enough to work closely with him, Tony is not only a world-leading scientist but also a trusted mentor, colleague, and friend. His optimism, curiosity, and gentle humour continue to enrich our community.

This Special Issue brings together the work of many of Tony’s long-standing collaborators, friends, and trainees. We are honoured to include contributions from distinguished international experts who have worked with Tony over the decades, including Lidietta Giorno (Italy), Xianhua Wang (China), Ranil Wickramasinghe and Andrew Zydney (USA), Chung-Hak Lee (Korea), Robert Field (UK), Matthias Kraume (Germany), Rong Wang (Singapore), and Joao Crespo (Portugal).

The papers collected in this Special Issue reflect Tony’s broad and enduring interests across the membrane field. Together, they span the full spectrum from fundamental science to applied research—an approach that Tony has championed throughout his career. Readers will encounter cutting-edge work on membrane material modification for diverse applications, ranging from pesticide degradation and environmental remediation to innovative strategies for bacterial quorum quenching. Several contributions advance our understanding of membrane fouling through new characterization approaches and diagnostic tools, continuing a theme to which Tony has contributed profoundly. Other papers examine membrane performance and integration in an impressively wide range of practical applications, including water treatment, municipal and industrial wastewater reuse, desalination, and even emerging uses within the health and biomedical sectors. This breadth of topics not only illustrates the dynamism of today’s membrane research but also mirrors Tony’s own expansive curiosity, multidisciplinary collaborations, and lifelong commitment to solutions that serve society.

As guest editors, we are delighted to present this Special Issue in honour of Tony. We hope it serves not only as a celebration of his remarkable career but also as an inspiration for future generations of membrane scientists. Tony has shown us what it means to pursue excellence with integrity, to build community through collaboration, and to advance science with purpose and humility. It is our privilege to honour him through this collection.

